# Inaugural Editorial

**DOI:** 10.48130/FR-2021-0001

**Published:** 2021-01-16

**Authors:** Hairong Wei

**Affiliations:** 1 Editor-in-Chief, Forestry Research; 2 Professor, Michigan Technological University

Forests provide not only timber, pulp, biofuel, food, and various industrial biomaterials for humans, but also habitats, shelters and forages for animals. Forests also provide a wide range of ecosystem services, for example, provisioning services (e.g. clean water and fresh air), regulating services (e.g. flood and erosion control, water and air purification, ground water recharge, and climate abnormality control), cultural services (e.g. recreational, aesthetic, and spiritual benefits), and supporting services (e.g. soil formation), which are vital in restoring and maintaining biodiversity in terrestrial ecosystem. Finally, forests play a leading role in the global cycling of energy, carbon, water and nutrients. To give you an idea of how important of a role forests play, it is estimated that forests store a trillion tons of the world’s carbon, equivalent to 42% of all CO_2_-emissions from human activity in the pre-industrial era (https://www.theworldcounts.com/). However, due to the global population explosion and increasingly intensified human activity in the last 60 years, atmospheric CO_2_ has escalated from 315 ppm to 415 ppm and is projected to be steadily elevated to 515 ppm by 2080, which will exacerbate global warming and climate catastrophe, and eventually, ecological disasters. Current studies have shown that prohibiting deforestation can reduce 10–15% of CO_2_ emissions, while promoting forestation will further reduce CO_2_. As the arctic permafrost (about 1.6 trillion tons of CO_2_) is thawing, forests will play an increasing role in combating global warming and climate change. For the past millennia, the livelihood of humans has largely depended on forest resources, but these resources are not inexhaustible. It is our responsibility to study their productivity, ecological functions, and their potential threats; we need to cultivate and advance our forestry research to ensure our forest resources are sustainable.

Because of the critically important functions of forest trees, forestry research has made considerable progress towards meeting escalating human demands, and combating global warming and ecosystem deterioration. However, some physiological characteristics and intrinsic properties of forest trees, such as large body sizes, long juvenile periods, long lifespans, recalcitrance to tissue culture, and genetic transformation, make it extraordinarily difficult to develop efficient experimental systems. As a result, forestry research generally has fallen behind that of model plants and important agricultural crops. As such, manuscripts on tree research are frequently depreciably evaluated by some journals with broad audiences. However, I have seen forestry research burgeoning in recent decades and I am optimistic this will continue in the coming years because: (1) the traditional forestry research has started to embrace big data and data mining technologies, and evolved in parallel with natural resource management. As a consequence, forestry research will become increasingly comprehensive and interesting; (2) the research focused on plant genomics has already shifted from simple model plant species (e.g. *Arabidopsis thalianas*) to staple crops to forest trees, rendering us unprecedented opportunity to elucidate complex traits controlled by complicated genomes; and (3) as global warming becomes increasingly severe, more funding resources will likely be deployed in supporting forestry research.

As output of basic forestry research rapidly increases, a dedicated journal is needed, and I am excited to introduce *Forestry Research (ForRes,* pronounced as /ˈfərrɪs/), a new international, open-access journal with rigorous editorial screening and assessment processes (http://www.maxapress.com/journal/forres).

The mission of *ForRes* is to provide the best service for your publishing needs, foster academic exchange, and to disseminate your novel findings across all forestry and related communities around the world. We aim to build *ForRes* into a flagship journal in basic forestry research. The specific aims of the journal are: (1) to become an open-source archive for storing and accessing top-notch scholarly works; (2) to become a highly trusted source of innovative information and technologies; (3) to become a much-needed platform for promoting worldwide academic exchange and knowledge dissemination in forestry and related disciplines.

The journal will publish original research articles, methodologies, reviews, and perspectives in one or multiple fields in forest science. The scope spans from molecules to populations to ecosystems, which include, but are not limited to: (1) genome and pan-genome analysis; (2) OMIC data and analysis; (3) biotechnology; (4) development; (5) genetics and breeding; (6) epigenetics; (7) biochemistry; (8) wood science; (9) tree physiology and ecophysiology; (10) ecology and evolution; (11) biometrics and inventory; (12) silviculture and forest management; (13) soil science; (14) abiotic and biotic stresses; (15) nitrogen-fixation; (16) hydrology; (17) forests and human interaction; (18) methodologies. In addition, *ForRes* will embrace new and emerging trends quickly by periodically publishing special issues.

*ForRes* is also committed to providing the best service to our authors and readers. All papers will be critically assessed by our editors and reviewers in a timely manner. Manuscripts that have been reviewed in other highly impactful journals will automatically enter an accelerated review process if the reviewers’ comments from other journals are also included. We will provide helpful feedback on rejected papers. At *ForRes*, we will work with our editorial board members, authors, readers, and public audience to expand and share your research through events and marketing. For example, *ForRes* will host webinars, workshops, and conferences on different topics with leading scientists in different fields, our editorial board members, and our top authors. We will encourage participants to submit manuscripts of Reviews, Methods, or Perspectives either individually or collaboratively. The editorial board will also provide training opportunities for the *ForRes* community − particularly graduate students and postdoctoral researchers − on research, publishing rudiments and essentials, and ethics of research, which will help advance forestry research and secure an influx of top-quality manuscripts for *ForRes*.

As the Editor-in-Chief, along with our editors, authors, readers, and the publishing community, I will make sure we will enforce the highest ethical standards in research and publishing. *ForRes* will strictly follow Committee on Publication Ethics (COPE) guidelines.

In a data-driven era of discovery, forestry professionals, regardless of their fields, will increasingly contribute, share, and reuse scientific big data. I wish to ensure the community that *ForRes* will embrace public data storage and sharing, because such data sharing and reuse will further stimulate and advance forestry research. We will invite experts for trainings and to guest-edit special issues related to big data acquisition, analysis, and mining. *ForRes* is published under the terms of the Creative Commons Attribution (CC BY) 4.0 License because we are committed to open science. We believe free access to scientific knowledge is the greatest contribution to the global humanity and environmental sustainability.

I would like to take this opportunity to express my sincerest gratitude to all passionate members who have volunteered to be a part of the *ForRes* Editorial Board and to serve the forestry research community worldwide. I will rely heavily on them not only in processing each manuscript, but also suggesting and organizing special issues in their fields of expertise. With the continuous and rapid development of *ForRes*, I will recruit more Associate Editors, especially those with expertise in emerging frontiers and interdisciplinary fields. If you are interested in joining the *ForRes* team as an Associate Editor, please contact me.

I wish to close this inaugural editorial by inviting you − forestry faculty, scientists, postdoctoral researchers, and graduate students − to submit your exciting research findings to *ForRes* and to accept invitations to provide reviews. Fueled by both passion and obligation, with your strong support, we are fully committed to developing this new community journal into a highly impactful, global journal that we can call a professional home and community in basic forestry research.

